# Estimation and Classification of NLFM Signals Based on the Time–Chirp Representation

**DOI:** 10.3390/s22218104

**Published:** 2022-10-22

**Authors:** Ewa Swiercz, Dariusz Janczak, Krzysztof Konopko

**Affiliations:** Faculty of Electrical Engineering, Bialystok University of Technology, 15-351 Bialystok, Poland

**Keywords:** NLFM signal classification, NLFM signal estimation, cubic phase function, multiclass classification, instantaneous frequency rate

## Abstract

A new approach to the estimation and classification of nonlinear frequency modulated (NLFM) signals is presented in the paper. These problems are crucial in electronic reconnaissance systems whose role is to indicate what signals are being received and recognized by the intercepting receiver. NLFM signals offer a variety of useful properties not available for signals with linear frequency modulation (LFM). In particular, NLFM signals can ensure the desired reduction of sidelobes of an autocorrelation (AC) function and desired power spectral density (PSD); therefore, such signals are more frequently used in modern radar and echolocation systems. Due to their nonlinear properties, the discussed signals are difficult to recognize and therefore require sophisticated methods of analysis, estimation and classification. NLFM signals with frequency content varying with time are mainly analyzed by time–frequency algorithms. However, the methods presented in the paper belong to time–chirp domain, which is relatively rarely cited in the literature. It is proposed to use polynomial approximations of nonlinear frequency and phase functions describing signals. This allows for applying the cubic phase function (CPF) as an estimator of phase polynomial coefficients. Originally, the CPF involved only third-order nonlinearities of the phase function. The extension of the CPF using nonuniform sampling is used to analyse the higher order polynomial phase. In this paper, a sixth order polynomial is considered. It is proposed to estimate the instantaneous frequency using a polynomial with coefficients calculated from the coefficients of the phase polynomial obtained by CPF. The determined coefficients also constitute the set of distinctive features for a classification task. The proposed CPF-based classification method was examined for three common NLFM signals and one LFM signal. Two types of neural network classifiers: learning vector quantization (LVQ) and multilayer perceptron (MLP) are considered for such defined classification problem. The performance of both the estimation and classification processes was analyzed using Monte Carlo simulation studies for different SNRs. The results of the simulation research revealed good estimation performance and error-free classification for the SNR range encountered in practical applications.

## 1. Introduction

In this paper, an approach based on the time–chirp (T−Ω) transform used for the estimation and classification of signals with nonlinear frequency modulation (NLFM) has been presented. The chirp rate (Ω), also called the instantaneous frequency rate (IFR), is the signal phase acceleration and can be calculated as the time derivative of a frequency function. The analysis and processing of NLFM signals are exploited in a wide range of applications for example in Electronic Support Measures/Electronic Intelligence (ESM/ELINT), Electronic Warfare (EW), Electronic Reconnaissance (ER) systems, as well as in passive bistatic radar (PBR) [1]. Modern electronic intelligence and electronic support are designed to automatically distinguish the modulation type of an intercepted radar signal, which can be utilized in early warning systems or give more information about hostile radars [2,3,4,5,6,7]. Passive bistatic radar uses emissions from communications, broadcast, or radionavigation transmitters instead of dedicated, cooperative radar transmitters. The transmitted waveforms are not explicitly designed for passive radar purposes. Therefore, knowledge about the received signal is crucial in the ability of waveform recognition and reconstruction. New sources of target illumination in passive radars are constantly being searched. Solutions with using the 5G cellular network as a source of illumination in a passive radar system have recently appeared [8]. The NLFM waveform for synthetic aperture radar (SAR) applications is important for improving spaceborne SAR image quality and reducing system costs [9,10]. The NLFM waveform was also proposed for active sonars [11].

The NLFM signal can be synthesized in different ways in order to obtain desired properties by shaping the power spectral density (PSD). Due to nonlinear frequency modulation, such signals can achieve the desired PSD and desired autocorrelation function with reduced sidelobes compared to LFM signals [12,13,14]. In the case of narrowband signals, the Doppler offset may be miscalculated in the narrowband ambiguity function [15,16]. The potential advantage of NLFM is its Doppler shift tolerance. These properties make NLFM signals very attractive especially for radar applications. Generally, synthesis of signals with limitation of the required level of AC-sidelobes and desired power spectral density is a great challenge. These conditions require multi-objective optimization approach with strong constraints and high computational load and may result in inconsistent requirements [11,15,17,18,19,20]. Appropriate NLFM chirps achieving the desired shape of the power spectrum have been suggested as a solution to this problem. Signals for which parameterized nonlinear frequency modulations satisfy the desired spectral properties, achieving low level sidelobes, have been proposed by a lot of authors. Signal models presented by Collins and Atkins [11], Pirce [21] or Yue and Zhang [22] are very popular and are also the subject of analysis in this paper.

A special class of NLFM signals, which enables the shaping of the PSD and obtains the desired AC function, is polynomial phased signals (PPS). This kind of nonlinear signal plays a significant role in modern radar systems, including SAR, ISAR and OTHR systems, as well as in sonars, biomedicine, machine engine testing, etc., especially in the ISAR, where due to target movement or extreme target maneuvers, the radar returns contain mainly PPS components, possibly with high-order phase terms [23,24]. The simplest type of a signal with a polynomial phase is a signal with linear frequency modulation. Unfortunately, the PSD of an LFM signal is approximately rectangular and after matched filtering (MF) the peak-to-sidelobe ratio (PSLR) is rather low, reaching about 13.3 [dB]. Therefore, NLFM signals are considered to be a good alternative to LFM signals.

Modern radar systems, especially surveillance systems, can emit a pulse train with inter-pulse and intra-pulse complex modulation, including both linear and nonlinear frequency modulation. If such radars operate in the dense, hostile electromagnetic environment, intercepted signals should be recognized or classified by means of spectrum-sensing systems such as ELINT, ER and EW. The main problem of recognition and estimation of intercepted signals is the determination of the modulation type and its parameters. If any information is not available, it is reasonable to assume a phase polynomial for such signals with a sufficiently high order of polynomial nonlinearity. Even parameterized nonlinearities, which usually have a complex analytical description, can be approximated by polynomial form with a sufficiently high order to obtain a simpler description for further analysis. In this paper, the polynomial approximation of the NLFM models has been evaluated for estimation and classification purposes. The set of selected coefficients of polynomial approximation is suggested as distinctive features allowing the identification of a type of unknown emission by means of classification. This approach requires a database containing a set of nonlinearity types. In this paper, the estimation of the PPS parameters as well as identification by classification of other type of nonlinearities are considered.

The general description of nonstationary signals embedded in noise can be presented as follows:

(1) $$s\left(t\right)=A\left(t\right)e^{j\phi (t)}+s_{n}\left(t\right)$$

where ϕ(t) is the phase of the signal, A(t) is the amplitude of the signal and $s_{n}\left(t\right)$ is white Gaussian noise.

There is an unambiguous relationship between frequency function f(t) (the instantaneous frequency (IF)) and the instantaneous phase function ϕ(t) of a nonstationary signal s(t) through the differentiation operation:

(2) $$f\left(t\right)=\frac{1}{2\pi }\frac{d\phi (t)}{dt}$$

Knowledge of the IF function automatically determines the phase function and vice versa. It seems natural to use time–frequency (T-F) distributions for IF analysis and estimation of NLFM signals. However, the most known quadratic T-F distributions, such as the pseudo Wigner–Ville distribution and the Choi–Williams distribution, contain cross-term components, and the estimation of the instantaneous frequency modulation is performed with unacceptable accuracy. Therefore, they are practically useless [2]. For analysis of the PPS, the high order ambiguity function (HAF) or the product HAF (PHAF) seems to be attractive. In these distributions, the phase-differentiation (PD) operation is repeated many times until a single complex sinusoidal signal is obtained [25,26]. The frequency of the obtained sinusoidal signal indicates the highest order PPS coefficient. Next, the original signal is dechirped with the use of this PPS coefficient. The remaining parameters are estimated by repeating the same procedure. Although these methods provide good accuracy, they suffer from high computational burden and error propagation during dechirping operations. Therefore, they seem to be useless for NLFM.

This paper deals with the effective estimation of the PPS of higher order. The proposed method is based on the concept of nonlinear sampling and the cubic phase function distribution (CPF) developed on the time–chirp (T−Ω) plane [27]. The CPF distribution turned out to be effective in parameter estimation of the quadratic frequency modulation signals [28]. In this paper, the CPF is proposed to extract distinctive features of parameterized nonlinearities approximated by a polynomial useful in classification. Nonlinearities within the paper are replaced by the polynomial form containing sufficient information to classify NLFM signals. Although the CPF was originally designed for the estimation of the third order PPS, in this paper, the CPF method is used for estimation of the sixth order PPS.

The classification of NLFM using the set of distinctive properties obtained from the CPF method on the (T−Ω) plane is presented in the next part of the paper. The classification process comprises three types of nonlinearities typical for radar applications. The classification of NLFM signals is not trivial, especially for signals with abrupt frequency changes, and requires advanced systems such as neural networks to achieve high classification efficiency [29,30,31,32]. Generally, due to the ability of self-learning and adaptability, neural networks can outperform other classification approaches. Two neural classifiers based on learning vector quantization (LVQ) and multilayer perceptron (MLP) networks have been used and compared. Other concepts of LFM and NLFM classification based on neural networks can be found in [33,34,35]. The neural network classification, considered in this paper, also takes into account the classification between NLFM and LFM signals [36].

The paper is organized as follows: Section 2 presents selected NLFM signals used in radar and sonar and discusses the use of their polynomial approximations. Section 3 discusses various methods of IF estimation and describes the proposed IF estimator based on the cubic phase function, while Section 4 presents the results of simulation studies on the quality of the proposed IF estimator. Section 5 proposes the use of the CPF-based estimator of phase polynomial coefficients in the classification of the NLFM signals and presents the results of simulation tests. Section 6 presents the conclusions.

## 2. Nlfm Signals and Their Polynomial Approximations

In this section, some selected examples of nonlinear functions used for the generation of NLFM signals for radar and echolocation systems are presented. Then, using the Taylor expansion of these functions, some aspects related to the accuracy of the polynomial approximation are illustrated and discussed.

Currently, NLFM signals are mainly developed for use in radar and sonar technologies. To present the proposed estimation and classification methods, three representative NLFM functions have been selected and presented by Formulas (3)–(7). The functions and their parameters were designed by their authors to minimize the sidelobes of the autocorrelation function.

The NLFM signal developed by Collins and Atkins [11] consists of a linear and nonlinear part. The nonlinearity impact is determined by two parameters α and γ:

(3) $$f_{1}\left(t,\alpha ,\gamma \right)=\frac{B}{2}\left[\frac{\alpha \tan(2\gamma t/T)}{\tan(\gamma )}+\frac{2(1-\alpha )t}{T}\right];\;-\frac{T}{2}\leq t\leq \frac{T}{2}$$

where *B* is the signal bandwidth, *T* is the time duration of the pulse, and α is the parameter that defines the weight between the linear and nonlinear part, while the parameter γ affects the intensity of the nonlinear part.

Parameter values that minimize the sidelobes of the autocorrelation function are α=0.52 and γ=1.47 [11].

The NLFM signal proposed by Price [21] also consists of a linear part represented by the $B_{L}$ parameter and a nonlinear part controlled by $B_{C}$:

(4) $$f_{2}\left(t,B_{L},B_{C}\right)=\frac{t}{T}\left(B_{L}+\frac{B_{C}}{\sqrt{1-4t^{2}/T^{2}}}\right).$$

In the paper, the following form of Formula (4), explicitly showing the signal bandwidth *B* [12], is used:

(5) $$f_{2}\left(t,B_{l},B_{c}\right)=B\frac{t}{T}\left(B_{l}+\frac{B_{c}}{\sqrt{1-4t^{2}/T^{2}}}\right),$$

where the parameters: $B_{l}=B_{L}/B$ and $B_{c}=B_{C}/B$, with values $B_{l}=0.561105$ and $B_{c}=0.23799$ minimizing sidelobes [12], and the time interval −0.45T≤t≤0.45T covering bandwidth *B*.

The NLFM signal proposed by Yue and Zhang [22] is given by the formula:

(6) $$f_{3}\left(t,k_{1},k_{2}\right)=Bk_{1}\tan\left(k_{2}\frac{t}{T}\right);\;-\frac{T}{2}\leq t\leq \frac{T}{2}.$$

where the parameters $k_{1}$ and $k_{2}$ that minimize the sidelobes take values: $k_{1}=0.1171$ and $k_{2}=2.607$.

In the presented analyses, the *LFM* signal is also used. It has the same bandwidth *B* and the pulse duration *T* as NLFM signals. The frequency of the *LFM* signal is described by the following formula:

(7) $$f_{LFM}\left(t\right)=\frac{B}{T}t;\;-\frac{T}{2}\leq t\leq \frac{T}{2}.$$

Nonlinear, continuous functions $f_{1}\left(t,\alpha ,\gamma \right)$, $f_{2}\left(t,B_{l},B_{c}\right)$, $f_{3}\left(t,k_{1},k_{2}\right)$ determined on the closed interval:$-\frac{T}{2}\leq t\leq \frac{T}{2}$, according to the Weierstrass approximation theorem, can be approximated with desired accuracy using polynomial functions of a sufficiently high order. The approximation error for a particular nonlinear function depends on the order of the polynomial.

The proposed estimation and classification method is based on simplified polynomial models of nonlinear functions that describe the frequency and phase of the NLFM signals. An important issue is the selection of the order of the approximating polynomial. In the paper, this issue is analyzed using the Taylor series approximation, which makes it possible to obtain an analytical description of the signal frequency and phase as a function of time.

Examples of the approximation of the nonlinear functions $f_{1}\left(t,\alpha ,\gamma \right)$, $f_{2}\left(t,B_{l},B_{c}\right)$, $f_{3}\left(t,k_{1},k_{2}\right)$ are presented by (8)–(10). Polynomials of the fifth order were calculated using the Taylor expansion around the time point *t* = 0:

(8) $$f_{1}^{Ta}\left(t,\alpha ,\gamma \right)=\frac{B}{T}\left(1-\alpha +\frac{\alpha \gamma }{\tan(\gamma )}\right)t+\frac{4B\alpha \gamma^{3}}{3T^{3}\tan\left(\gamma \right)}t^{3}+\frac{32B\alpha \gamma^{5}}{15T^{5}\tan\left(\gamma \right)}t^{5}+R_{f1}\left(t,\alpha ,\gamma \right)$$

(9) $$f_{2}^{Ta}\left(t,B_{l},B_{c}\right)=\frac{B(B_{c}+B_{l})}{T}t+\frac{2BB_{c}}{T^{3}}t^{3}+\frac{6BB_{c}}{T^{5}}t^{5}+R_{f2}\left(t,B_{c},B_{l}\right)$$

(10) $$f_{3}^{Ta}\left(t,k_{1},k_{2}\right)=\frac{Bk_{1}k_{2}}{T}t+\frac{Bk_{1}{k_{2}}^{3}}{3T^{3}}t^{3}+\frac{2Bk_{1}{k_{2}}^{5}}{15T^{5}}t^{5}+R_{f3}\left(t,k_{1},k_{2}\right)$$

where $R_{f1}\left(t,\alpha ,\gamma \right)$, $R_{f2}\left(t,B_{c},B_{l}\right)$ and $R_{f3}\left(t,k_{1},k_{2}\right)$ are reminder terms.

**Figure 1:**
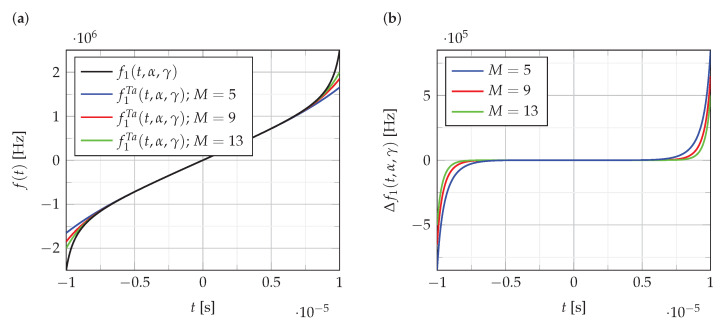
The accuracyof nonlinear frequency approximations, (**a**) the function $f_{1}\left(t,\alpha ,\gamma \right)$ and its Taylor polynomial approximations of the order M∈{5,9,13}, (**b**) the error of approximations.

Figure 1 illustrates a change in the accuracy of $f_{1}^{Ta}\left(t,\alpha ,\gamma \right)$ approximation depending on the polynomial order. Figure 1a shows the function $f_{1}\left(t,\alpha ,\gamma \right)$ and its polynomial approximations of order M∈{5,9,13}. Figure 1b shows the error $\Delta f_{1}\left(t,\alpha ,\gamma \right)$ of these approximations.

(11) $$\Delta f_{1}\left(t,\alpha ,\gamma \right)=f_{1}\left(t,\alpha ,\gamma \right)-f_{1}^{Ta}\left(t,\alpha ,\gamma \right)$$

As can be seen from the analysis of the results presented in Figure 1, the error of polynomial approximation for the order M>5 slowly decreases. Similar results are obtained for the approximation of the functions $f_{2}\left(t,B_{l},B_{c}\right)$ and $f_{3}\left(t,k_{1},k_{2}\right)$.

Based on the relationship (2), it is possible to calculate the analytical functions (12)–(15) that describe the instantaneous phase of the NLFM and LFM signals:

(12) $$\phi_{1}\left(t,\alpha ,\gamma \right)=\pi B\left[\frac{\alpha T\ln({\tan(2\gamma t/T)}^{2}+1)}{4\gamma \tan(\gamma )}+\frac{\left(1-\alpha \right)t^{2}}{T}\right]+\phi_{1}^{0},$$

(13) $$\phi_{2}\left(t,B_{l},B_{c}\right)=\frac{\pi }{2}B\left(\frac{2B_{l}}{T}t^{2}-B_{c}\sqrt{T^{2}-4t^{2}}\right)+\phi_{2}^{0},$$

(14) $$\phi_{3}\left(t,k_{1},k_{2}\right)=\frac{\pi BTk_{1}}{k_{2}}\ln\left({\tan(k_{2}\frac{t}{T})}^{2}+1\right)+\phi_{3}^{0},$$

(15) $$\phi_{LFM}\left(t\right)=\frac{\pi B}{T}t^{2}+\phi^{0},$$

where $\phi_{1}^{0}$, $\phi_{2}^{0}$, $\phi_{3}^{0}$, $\phi^{0}$ are initial phase values, while the time interval is is $-\frac{T}{2}\leq t\leq \frac{T}{2}$.

The Taylor polynomial approximation is calculated around the time moment t=0 nd phase value: ϕ(0), as the phase function is symmetrical with respect to this point. Taylor approximations of the phase with polynomials of the sixth order are presented by (16)–(18).

(16) $$\phi_{1}^{Ta}\left(t,\alpha ,\gamma \right)=\frac{\pi B}{T}\left(1-\alpha +\frac{\alpha \gamma }{\tan(\gamma )}\right)t^{2}+\frac{2\pi B\alpha \gamma^{3}}{3T^{3}\tan\left(\gamma \right)}t^{4}+\frac{32\pi B\alpha \gamma^{5}}{45T^{5}\tan\left(\gamma \right)}t^{6}+R_{\phi 1}\left(t,\alpha ,\gamma \right)$$

(17) $$\phi_{2}^{Ta}\left(t,B_{l},B_{c}\right)=-\frac{\pi }{2}BB_{c}T+\frac{\pi B(B_{c}+B_{l})}{T}t^{2}+\frac{\pi BB_{c}}{T^{3}}t^{4}+\frac{2\pi BB_{c}}{T^{5}}t^{6}+R_{\phi 2}\left(t,B_{c},B_{l}\right)$$

(18) $$\phi_{3}^{Ta}\left(t,k_{1},k_{2}\right)=\frac{\pi Bk_{1}k_{2}}{T}t^{2}+\frac{\pi Bk_{1}{k_{2}}^{3}}{6T^{3}}t^{4}+\frac{2\pi Bk_{1}{k_{2}}^{5}}{45T^{5}}t^{6}+R_{\phi 3}\left(t,k_{1},k_{2}\right)$$

where $R_{\phi 1}\left(t,\alpha ,\gamma \right)$, $R_{\phi 2}\left(t,B_{c},B_{l}\right)$, $R_{\phi 3}\left(t,k_{1},k_{2}\right)$ are reminder terms.

**Figure 2:**
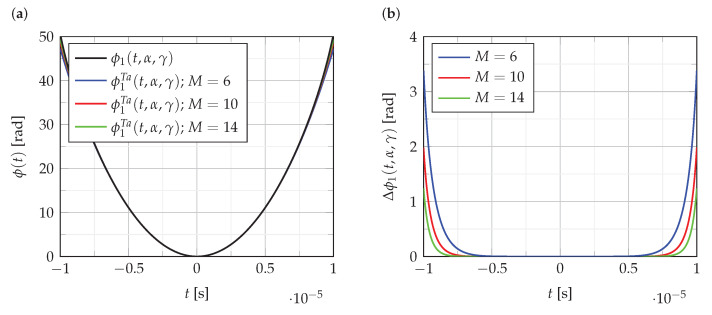
The accuracyof phase function approximations, (**a**) the function $\phi_{1}\left(t,\alpha ,\gamma \right)$ and its Taylor polynomial approximations of order M∈{6,10,14}; (**b**) the error of approximations.

The influence of polynomial order on the accuracy of the polynomial approximation of the phase function $\phi_{1}\left(t,\alpha ,\gamma \right)$ is presented in Figure 2. Figure 2a shows the function $\phi_{1}\left(t,\alpha ,\gamma \right)$, and its polynomial approximations of order M∈{6,10,14}. Figure 2b shows the error $\Delta \phi_{1}\left(t,\alpha ,\gamma \right)$ of these approximations:

(19) $$\Delta \phi_{1}\left(t,\alpha ,\gamma \right)=\phi_{1}\left(t,\alpha ,\gamma \right)-\phi_{1}^{Ta}\left(t,\alpha ,\gamma \right)$$

As can be seen from the analysis of the results presented in Figure 2, the error of polynomial approximation of phase function is quite small compared to phase value. Similar results are obtained for the approximation of the functions $\phi_{2}\left(t,B_{l},B_{c}\right)$ and $\phi_{3}\left(t,k_{1},k_{2}\right)$.

In the paper, the polynomial approximation of a phase function has been analyzed to classify NLFM signals. The nonlinear change in the frequency of analyzed signals is described by the odd function, which results in an odd order of approximating polynomial. Taking into account this feature and the relationship (2), the polynomial approximation of the phase function is of an even order. In our case, after preliminary investigation, we decided that the order of the polynomial approximation of the phase function can be limited to M=6. The set of selected coefficients of the approximating polynomial is chosen as a set of distinctive features in the proposed method of classification of NLFM signals. The sixth order of the approximating polynomial seems sufficient to constitute a set of distinctive features. Although the selected order of the approximating polynomial does not guarantee a perfect polynomial fitting, especially on signal parts with abrupt nonlinear changes, it allows for effective classification with the low computational load.

## 3. The CPF-Based Estimator of the IFR

The recognition of signals with nonlinear frequency modulation is usually performed with the use of estimation of the instantaneous frequency of the signal. There are many methods for analyzing nonlinear frequency functions, including all distributions of the group belonging to the Cohen class. These are, among others, time–frequency distributions such as the Wigner–Ville distribution, the Choi–Wiliams distribution and the short-time Fourier transform (STFT). A large group of signals with a nonlinear frequency are PPS. Very good estimation results of the higher order PPS are obtained by the quasi-maximum likelihood (QML) method, which is an extended version of the STFT transformation [37,38,39]. Estimation of the IF is performed by the STFT. Coefficients of the PPS are obtained from the IF estimates using the classical polynomial regression. The QML method requires an additional refining procedure to improve the quality of coarse estimates of polynomial coefficients. The refining process consists of four steps: dechirping the received signal using coarse initial estimates of the polynomial phase parameters provided by the STFT, filtering through an M-point moving average (MA) filter combined with an M-fold decimation, polynomial phase estimation of the obtained signal by phase unwrapping and least squares estimation. The final estimates are calculated as a combination of estimates obtained in step 3 with the initial coarse estimates [40]. In the last step of the QML method, the optimal STFT window is searched by maximizing the quasi-ML function. Rather than directly searching through all parameters of phase polynomial, the maximum QML function is calculated for the estimates provided by STFT and polynomial regression. The QML is computationally exhaustive for higher order PPS because it requires multiple STFT calculations and multiple polynomial regression calculations, which are slow but precise processes. Therefore, it is desirable to search for a new transform with results comparable to the QML transform, but with less computational effort. A proposed alternative method of estimating the parameters of approximating polynomials is the CPF distribution defined for discrete signals.

Discrete signals of interest (i.e., PPS) $z_{r}\left(n\right)$ are characterized by a constant amplitude $b_{0}$ and phase ϕ(n) and are defined as:

(20) $$z_{r}\left(n\right)=z_{s}\left(n\right)+z_{w}\left(n\right)=b_{0}e^{j\phi (n)}+z_{w}\left(n\right),\;-\frac{N-1}{2}\leq n\leq \frac{N-1}{2},$$

where the phase function ϕ(n) is described by the M−th order polynomial with coefficients $a_{m}$ and $z_{w}\left(n\right)$ is Gaussian white noise with variance $\sigma^{2}$. The discrete phase function is specified by the following formula:

(21) $$\phi \left(n\right)=\sum_{m=0}^{M}a_{\phi m}n^{m}$$

The CPF is defined as follows:

(22) $$CPF_{z_{r}}\left(n,\Omega \right)=\sum_{m=0}^{\frac{N-1}{2}}z_{r}\left(n+m\right)z_{r}\left(n-m\right)e^{-j\Omega m^{2}}$$

where Ω is the frequency rate.

The discrete time $t_{n}=nT_{s}$ resulting from sampling with the period $T_{s}$ and the discrete frequency rate Ω define a discrete grid on the time—frequency rate plane (n,Ω). Therefore, the discrete results of the estimation of phase polynomial coefficients may differ from the continuous case if the discrete grid is sparse. The estimate of IFR [28,41] for each point in time is obtained as follows:

(23) $$\hat{IFR}\left(n\right)=\underset{\Omega }{\mathrm{argmax}}CPF_{z_{r}}\left(n,\Omega \right)$$

The CPF presented in the literature is mainly used to estimate the parameters of a signal phase polynomial up to the third order [28,41,42]. However, according to the analysis presented in Section 2, the proposed classification approach requires estimation of the coefficients of the sixth order phase polynomial. In this paper, the extension of the CPF to analysis of a sixth order polynomial is considered. The proposed approach assumes that only one run of the CPF-based method is used to estimate coefficients of the phase polynomial of the required order. Having had the set of estimated phase polynomial coefficients, the set of frequency polynomial coefficients can also be calculated according to the relationship (2). The proposed method based on the CPF has a lower computational load than the QML and other commonly known methods dedicated to the analysis of signals in the frequency or time–frequency domains.

NLFM signals are most often defined by frequency functions, such as functions $f_{1}\left(t,\alpha ,\gamma \right)$, $f_{2}\left(t,B_{L},B_{C}\right)$ and $f_{3}\left(t,k_{1},k_{2}\right)$ given in Section 2 described by (3)–(6). Generating signals by means of Equation (1) requires the knowledge of the corresponding phase functions, which for the analyzed signals are represented by the functions $\phi_{1}\left(t,\alpha ,\gamma \right)$, $\phi_{2}\left(t,B_{L},B_{C}\right)$ and $\phi_{3}\left(t,k_{1},k_{2}\right)$ described by (12)–(14). The CPF algorithm, which processes the received noise-disturbed signal (20), estimates the coefficients of the discrete phase polynomial model. In the proposed classification method, these coefficients of the phase polynomial are used as a set of distinctive features. Classification methods are described in more detail in Section 5. Moreover, the estimation of the instantaneous frequency with the use of a discrete polynomial approximating the continuous frequency function is proposed. The coefficients of the approximating frequency polynomial are calculated from the coefficients of the phase polynomial obtained using CPF.

The proposed M−th order polynomial approximating frequency function f(n) is of the form:

(24) $$\hat{f(n)}=\sum_{m=0}^{M}a_{fm}n^{m},$$

where $a_{fm}$ are the coefficients of the frequency polynomial.

The coefficients of the frequency polynomial (24) can be obtained from phase polynomial coefficients using relationships (2) and take the following value:

(25) $$a_{fm}=\frac{\left(m+1\right)a_{\phi m+1}}{2\pi T_{s}}$$

where the coefficients $a_{\phi m}$ of the phase polynomial are obtained by the CPF.

Modification of the kernel of the classical CPF distribution (22) with the use of nonuniform sampling allows for higher order PPS decomposition. By sampling the signal at nonuniformly spaced time moments, the order of the PPS estimator can be lowered [43]. The kernel of the original CPF distribution (22) is as follows:

(26) $$K\left(z_{r},n\right)=z_{r}\left(n-m\right)z_{r}\left(n+m\right)$$

and the modified kernel takes the following form:

(27) $$K\left(z_{r},n\right)=z_{r}\left(n-\sqrt{Cm}\right)z_{r}\left(n+\sqrt{Cm}\right),\;m=0,1,\cdots ,\frac{N-2}{2}-\left|n\right|$$

where $\sqrt{Cm}$ defines the nonlinear sampling [44].

The proposed sampling allows the calculation of the CPF using the FFT method, which results in a significant reduction of the computational load.

If we consider the modified kernel for *n* = 0 and the signal model (2) processed by this kernel, a third order polynomial form is obtained with only even coefficients from the set of all coefficients of the sixth order phase polynomial, which is proposed as a polynomial approximation of considered nonlinearities:

(28) $$K\left(z_{r},0\right)=z_{r1}\left(m\right)=e^{j2\left(Ca_{2}m+C^{2}a_{4}m+C^{3}a_{6}m^{3}\right)}$$

where the parameter C=(N−1)/2 controls the sampling process, and $a_{2}$, $a_{4}$, $a_{6}$ are polynomial parameters related to phase polynomial coefficients from (21).

The kernel $K(z_{r},0)$ creates the signal with the polynomial phase of the 3rd order. Coefficients of such a polynomial can be efficiently computed using the CPF distribution (22). Parameters $2Ca_{2}$, $2C^{2}a_{4}$ and $2C^{3}a_{6}$ are related to $a_{\phi 2}$, $a_{\phi 4}$, $a_{\phi 6}$, respectively. Therefore, the estimation of the polynomial parameters can be performed directly by the CPF procedure:

(29) $${\hat{\Omega }}_{1}=arg\max_{\Omega }CPF_{z_{r}}\left(n_{1},\Omega \right)$$

(30) $${\hat{\Omega }}_{2}=arg\max_{\Omega }CPF_{z_{r}}\left(n_{2},\Omega \right)$$

It is natural to assume the parameter $n_{1}=0$, but parameter $n_{2}$ should be chosen to obtain a statistically optimal estimate of parameters. It strongly depends on the properties of analyzed signals. The ${\hat{\Omega }}_{1}$ and ${\hat{\Omega }}_{2}$ allow for calculating $\hat{a_{\phi 4}}$ and $\hat{a_{\phi 6}}$ according to Equations (31) and (32) [28]:

(31) $$\hat{a_{\phi 4}}=\frac{\Omega_{1}n_{2}-\Omega_{2}n_{1}}{4C^{2}\left(n_{2}-n_{1}\right)}$$

(32) $$\hat{a_{\phi 6}}=\frac{\Omega_{2}-\Omega_{1}}{12C^{3}\left(n_{2}-n_{1}\right)}$$

An estimate of $a_{2}$ from (28) is obtained by dechirping and finding the Fourier transform peak and $\hat{a_{\phi 2}}$ can be calculated as follows:

(33) $$\hat{a_{\phi 2}}=\frac{\hat{a_{2}}}{2C}$$

The kernel (28) is independent of the remaining parameters $a_{\phi 1}$, $a_{\phi 3}$, $a_{\phi 5}$ of the phase polynomial. The accuracy of the estimation process depends on the SNR, and operations above a certain SNR threshold are performed with acceptable accuracy.

**Figure 3:**
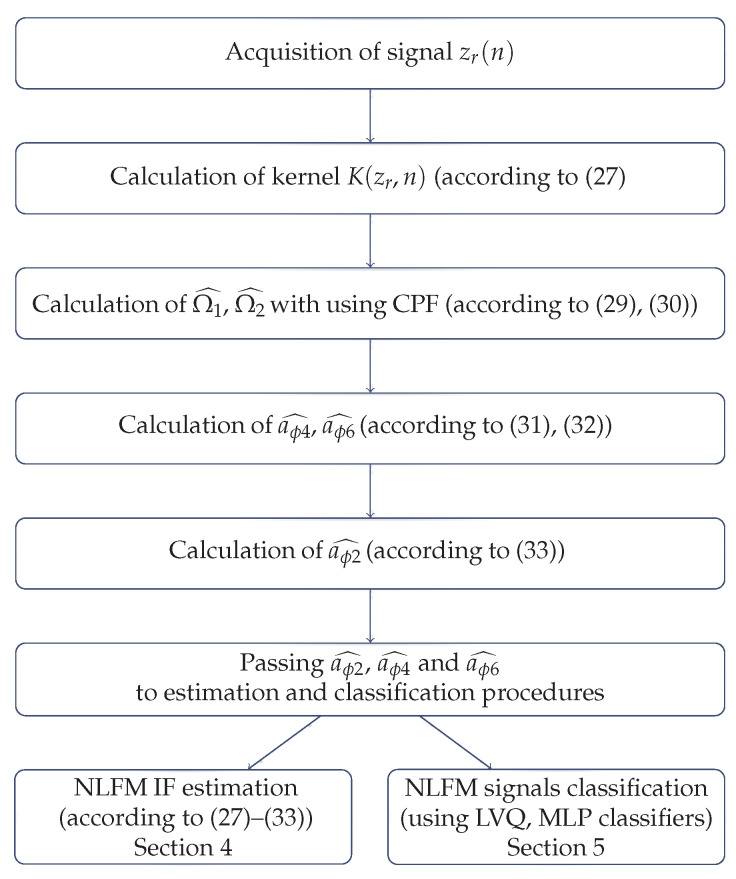
Flow diagram of the proposed method including estimation and classification.

The estimated parameters $a_{\phi 2}$, $a_{\phi 4}$ and $a_{\phi 6}$ are used for the estimation of the nonlinear frequency function and the signal classification process. The proposed method including estimation and classification is summarized in the flow diagram presented in Figure 3.

## 4. Frequency Estimation of NLFM Signals Based on CPF

Simulation investigations of the proposed algorithms were carried out for three waveforms NLFM and one LFM waveforms. The NLFM functions, marked as $f_{1}\left(t,\alpha ,\gamma \right)$, $f_{2}\left(t,B_{L},B_{C}\right)$ and $f_{3}\left(t,k_{1},k_{2}\right)$, are presented in Section 2 and are described by (3)–(6). Their specific parameters ensuring minimal sidelobes are presented there. The up-chirp LFM waveform $f_{LFM}\left(t\right)$ is described by Formula (15). The following time and bandwidth parameters for all signals were assumed: pulse duration $T=20\cdot 10^{-6}$ s. (with time frame: −T/2≤t≤T/2); sampling frequency $f_{s}=100$ MHz; signal bandwidth B=5 MHz. The signal noise was assumed to be complex Gaussian with variance depending on the SNR. To evaluate the proposed methods, $N_{sr}=500$ Monte Carlo simulations were carried out for each case.

For assumed sixth order phase polynomial and odd frequency functions, the estimation function takes the form:

(34) $$\hat{f(n)}=a_{f1}n+a_{f3}n^{3}+a_{f5}n^{5},$$

where $a_{f1}$, $a_{f3}$ and $a_{f5}$ are polynomial coefficients that can be calculated as follows:

(35) $$a_{f1}=\frac{a_{\phi 2}}{\pi T_{s}}$$

(36) $$a_{f3}=\frac{a_{\phi 4}}{\pi T_{s}}$$

(37) $$a_{f5}=\frac{a_{\phi 6}}{\pi T_{s}}$$

where $a_{\phi 2}$, $a_{\phi 4}$ and $a_{\phi 6}$ are the coefficients of the polynomial approximating phase obtained from the CPF.

The quality of the estimation of the instantaneous frequency of the signal can be assessed by the root mean square error (RMSE) determined according to the relationship:

(38) $$RMSE\left(k\right)=\sqrt{\frac{1}{N_{sr}}\left[\sum_{n=1}^{N_{sr}}{\left(\hat{f_{n}\left(k\right)}-f_{NLFM}\left(k\right)\right)}^{2}\right]}$$

where $N_{sr}$ is the number of simulation runs.

**Figure 4:**
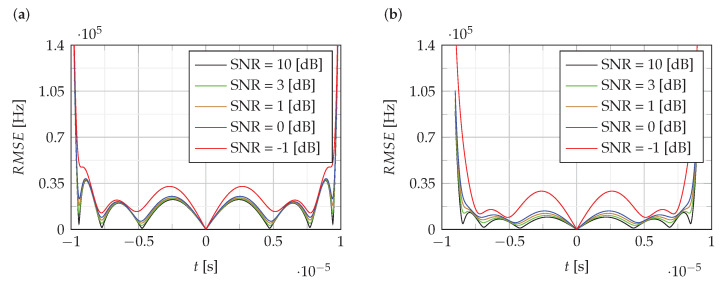
The RMSEof estimation of the instantaneous value of the frequency: (**a**) $f_{1}\left(t,\alpha ,\gamma \right)$, (**b**) $f_{2}\left(t,B_{l},B_{c}\right)$.

**Figure 5:**
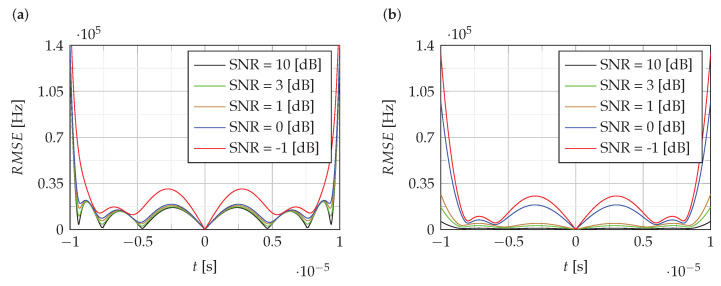
The RMSEof estimation of the instantaneous value of the frequency: (**a**) $f_{3}\left(t,k_{1},k_{2}\right)$; (**b**) $f_{LFM}\left(t\right)$.

Figure 4 and Figure 5 show the RMSE of the estimation of the instantaneous value of the frequency of NLFM signals obtained using the proposed CPF-based method. For comparison, Figure 5b shows the RMSE for the LFM signal.

The analysis of RMSE presented in Figure 4 and Figure 5 shows that the estimation error level is mainly around 0.5% of the bandwidth B, except for the edges where it increases to 5%.

Then, the mean square error (MSE) dependence on the SNR was determined for the estimates of the individual NLFM and LFM signals. The MSE was defined as follows:

(39) $$MSE\left(SNR\right)=\log_{10}\frac{1}{N_{sr}}\frac{1}{N_{sl}}\sum_{n=1}^{N_{sr}}\sum_{n=1}^{N_{sr}}{\left(\hat{f_{n}\left(k\right)}-f_{NLFM}\left(k\right)\right)}^{2}$$

where $N_{sr}$ is the number of simulation runs, and Nsl is the number of signal samples (signal length).

**Figure 6:**
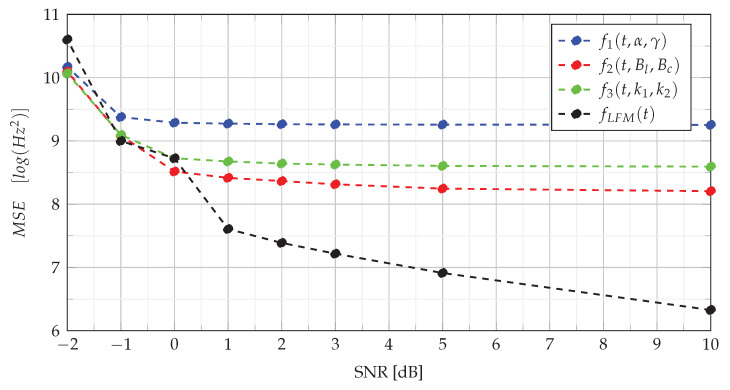
The MSEof estimation of the instantaneous frequency under various SNR conditions.

The MSE defined in this way allows for determining the estimation error for the entire signal (the entire pulse) and enabling the comparison of the obtained results with the results presented in the publication [2]. Figure 6 presents the MSE of estimation of the instantaneous frequency in various SNR conditions.

The analysis of the MSE of NLFM signals estimation presented in Figure 6 shows that the estimation error level of the NLFM signal depends on its type. The error remains constant at SNR≥0 dB and increases slightly for SNR=−1 dB. However, in the case of SNR≤−2 dB, the MSE increases significantly. On the other hand, the MSE for LFM is much lower than for NLFM, and the error decreases as the noise level decreases. Compared to other methods of estimation of the IF, the method based on CPF shows a similar estimation quality. For example, comparing the obtained results with those presented in [2], it can be noticed that, for LFM, the MSE obtained using the CPF method for SNR≥2 dB is smaller than for the QML method, which, according to [2], shows lower estimation errors than methods such as the backward finite difference (BFD) method, central finite difference (CFD) estimator, Kay estimator, estimators based on the Choi–Williams distribution (CWD) and pseudo-Wigner distribution (PWD). However, for a higher noise level, QML provides better results. In the case of the estimation of NLFM signals, the QML method comes up with a slightly lower MSE than the CPF method over the entire SNR range.

The complexity of the QML algorithm, in terms of the number of operations performed, depends on the assumed instantaneous frequency (IF) resolution in the procedure of searching the STFT maximum. Similarly, the complexity of the CPF algorithm strongly depends on the instantaneous frequency rate (IFR) resolution in the procedure of searching for the CPF maximum. These maximization operations are performed on two different planes i.e., time–frequency (T-F) and time–chirp rate (T-FR). This imposes different resolution requirements that must be applied when we calculate the maximum points for the STFT and the CPF in successive identical time moments. This affects the accuracy of the estimation, as well as the execution time of the algorithms. In the comparison, the experiments with the typical values of the procedure parameters in both algorithms showed the lower computational load for the CPF algorithm compared to the QML method several dozen times.

Despite the slightly lower quality of the estimation, considering the much lower computational load of the CPF method compared to the QML, it can be concluded that the CPF may be preferred in real-time applications.

## 5. Classification of Signals Based on Phase Polynomial Coefficiencies Obtained from CPF

The classification procedure presented in this paper concerns the problem of recognizing signal types with nonlinear frequency modulation. The main problem is to find a set of distinctive features that allow the received signals to be distinguished and classified into a class related to a specific emitter. Generally, three kinds of classification tasks are mainly used:

1. Binary classification—in this case, there are only two classes;
2. Multiclass classification—in this case, there are more than two classes, and the classifier can only report one of them as output;
3. Multilabel classification—in this case, the classifier is allowed to choose many answers. This type of classification can be simply considered as a combination of multiple independent binary classifiers.

The classification task considered in the paper can be associated with a multiclass classification, in which the class is defined by a specific type of nonlinearity of the frequency function. Therefore, it is the type of classification indicated in item 2 of the above three-point list.

Many different modifications of the multiclass classification method have been proposed in the literature [45,46,47]. The proposed method uses multiclass classification with a vector of features. The classification between three types of NLFM signals and one LFM signal described in Section 2 is considered. The feature vector is formed by aggregation of selected CPF coefficients describing the polynomial approximation of the considered phase functions.

The extracted features are processed by the classifier to select the most probable class. A supervised classification is considered, where the classes are known in advance and samples of the features describing each class are available. The feature vectors for individual nonlinearities can be treated as a pattern in the feature space. Therefore, classification carried out, especially by a neural network, is a problem of recognizing patterns [33,34]. In this paper, two types of neural network classifiers: learning vector quantization (LVQ) and multilayer perceptron (MLP) are considered. The LVQ neural network has been chosen because of its high ability to learn data classification, where similar input vectors are grouped into a region represented by the so-called coded vector (CV). LVQ can be applied directly to multiclass classification problems. LVQ is a supervised version of vector quantization. LVQ uses known target output classifications for each input pattern in supervised learning of the neural network. The input space of samples is covered by the “codebook vectors” (CVs) determined during the neural network learning stage. The LVQ neural network is built as a feedforward net with one hidden layer of neurons (the Kohonen layer), fully connected with the input layer and one output layer. During the training stage, the values of weights used to form the coded vectors are adjusted, according to the previously predefined input patterns. The distance $d_{i}$ of an input vector signed *x* to the weight vector $w_{i}$ of each node in the Kohonen layer is computed. The node of a particular class, which has the smallest distance to the presented input vector (for example the Euclidean distance), is declared to be the winner:

(40) $$d_{i}=∥w_{i}-x∥=\sqrt{\sum_{j=1}^{M}{\left(w_{ij}-x_{j}\right)}^{2}}$$

The weights will be moved closer to that class, which is expected as the winning class. Otherwise, they will be moved away. The classification after learning is relied on finding a Voronoi cell, specified by the CV with the smallest distance to the input vector and assigning it to a particular class.

**Figure 7:**
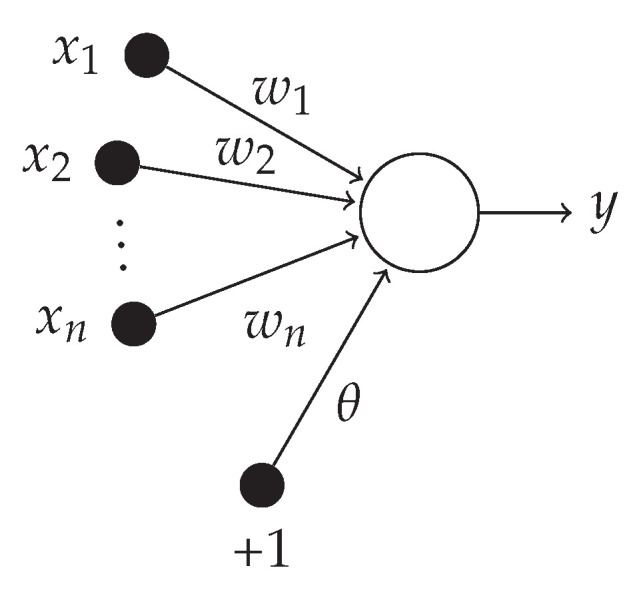
Single layer network with one output and *n* inputs.

The designed LVQ classifier contains 4N competing neurons with the logistic sigmoid function as an activation function, where *N* is the number of classes. The MLP is a fundamental type of neural network architecture with the ability to learn nonlinear models. The multi-layer perceptron (MLP) is a type of artificial neural network organized in several layers in which the flow of information takes place from the input layer to the output layer; therefore, it is a feedforward network. Each layer is made up of a variable number of neurons, and the neurons of the last layer (called the “output”) are the outputs of the entire system. A network of such perceptrons is termed a neural network of perceptrons. A perceptron with only an input and output layer is called a simple perceptron. A single layer feed-forward network consists of one or more output neurons *o*, each of which is connected with a weighting factor $w_{io}$ to all of the inputs *i*. The input of the neuron is the weighted sum of the inputs plus the bias term θ. A example of a single layer network with *n* inputs and one output is shown in Figure 7.

The output of the network is formed by the activation of the output neuron, which is some function of inputs:

(41) $$y=f\left(\sum_{i=1}^{N}w_{i}x_{i}+\theta \right)$$

The training of a neural network is the procedure of setting its weights. If there is one hidden layer, this one is a two layer perceptron. The aim of the supervised MLP network training is to achieve an appropriately small mean square error obtained in the Levenberg–Marquardt backpropagation procedure by adjusting weights. The complexity of the neural network classifier strongly depends on a number of neurons, which require an adjustment of their weighs at the learning stage. Therefore, the neural architecture should be as simple as possible.

For the assumed classification task, a simple single hidden layer MLP classifier with the number of neurons equal to 2N has been chosen.

**Figure 8:**
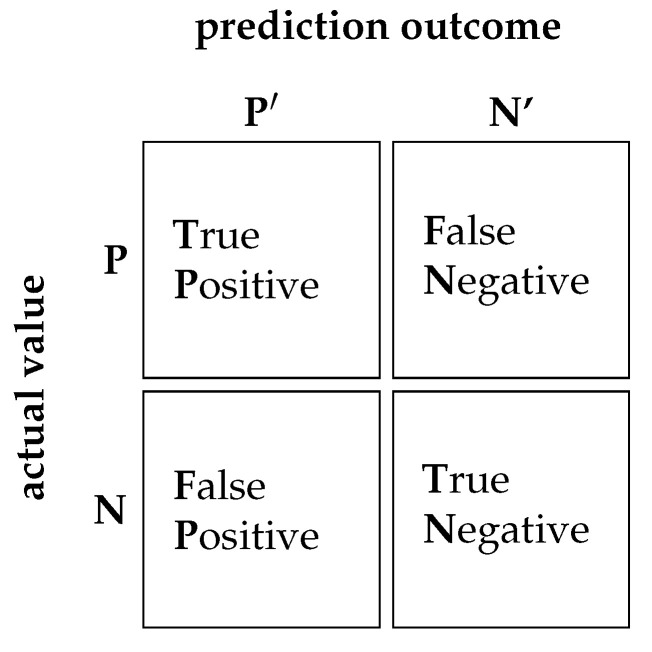
Interpretation of the binary confusion matrix.

Evaluation of the classification process requires appropriate quality criteria. The classification performance is usually visualized using the confusion matrix, which is a table summarizing true and false decisions. The matrix compares the actual types of objects with those predicted by the classifier. This issue can be easily presented on the example of binary classification. In this case, the 2×2 confusion matrix is formulated as shown in Figure 8.

The abbreviations TP, TN, FP and FN shown in Figure 8 denote:

- true positives (TP): the actual value is positive and the prediction is also positive;
- true negatives (TN): the actual value is negative and the prediction is also negative;
- false positives (FP): the actual value is negative, but the prediction is positive (type I error);
- false negatives (FN): the actual value is positive, but the prediction is negative (type II error).

It is difficult to compare the properties of different classifiers based on the confusion matrix alone. Therefore, a simpler description of the classification can be obtained using metrics calculated on the basis of data from the confusion matrix. Some common metrics [48] can be calculated as follows:

1. Accuracy (*ACC*)
  (42) $$ACC=\frac{TP+TN}{TP+TN+FP+FN}$$
2. Precision also known as positive predictive value (*PPV*)
  (43) $$PPV=\frac{TP}{TP+FP}$$
3. Sensitivity also known as recall, hit rate or true positive rate (*TPR*)
  (44) $$TPR=\frac{TP}{TP+FN}$$

The above metrics are defined similarly for multiclass classifiers. To evaluate the performance of the proposed multiclass model, the N×N confusion matrix is used, where *N* is the number of classes that describe the NLFM or LFM signals. The quality of the selected classifiers can be assessed by comparing the metrics calculated from their confusion matrix.

**Figure 9:**
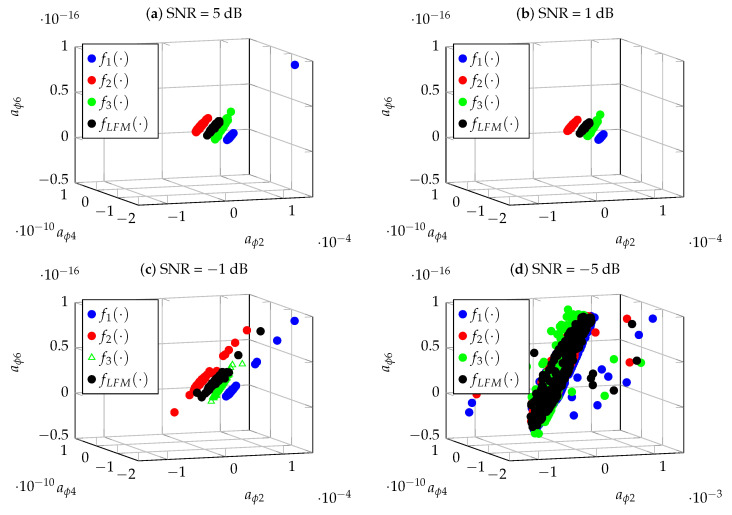
Example ofthe realizations of the coefficients $a_{\phi 2}$, $a_{\phi 4}$ and $a_{\phi 6}$ obtained by CPF for four signal classes in the case of (**a**) SNR=5 dB; (**b**) SNR=1 dB; (**c**) SNR=−1 dB; and (**d**) SNR=−5 dB.

The performance of the proposed method was evaluated with the use of the simulation experiment. The proposed CPF-based classification method was tested for three NLFM ($f_{1}\left(t,\alpha ,\gamma \right)$, $f_{2}\left(t,B_{l},B_{l}\right)$, $f_{3}\left(t,k_{1},k_{2}\right)$) and one LFM ($f_{LFM}\left(t\right)$) signal. Their specific parameters are presented in Section 2. The simulation parameters were assumed to be the same as in the case of simulation presented in Section 4, namely: pulse duration $T=20\cdot 10^{-6}$ s.; sampling frequency $f_{s}=100$ MHz; signal bandwidth B=5 MHz and Gaussian noise with variance depending on SNR. Thus, having four signals to recognize, the problem of 4-class classification is studied. The feature vector used in the classification process was formed by the coefficients ($a_{\phi 2}$, $a_{\phi 4}$, $a_{\phi 6}$) of the polynomial which is an approximate signal phase. The coefficients are determined by the proposed CPF method. Figure 9 shows an example of the realizations of the coefficients $a_{\phi 2}$, $a_{\phi 4}$ and $a_{\phi 6}$ obtained by CPF for four signal classes in the case of SNR=5 dB, SNR=1 dB, SNR=−1 dB and SNR=−5 dB. Each signal is marked with a different colour. The points in the figure represent the estimates of the coefficients $a_{\phi 2}$, $a_{\phi 4}$ and $a_{\phi 6}$ obtained in $N_{sr}=500$ realizations of individual signals.

The analysis of the spatial position of the coefficients $a_{\phi 2}$, $a_{\phi 4}$ and $a_{\phi 6}$ shown in Figure 9 shows that, in the case of SNR=5 dB and SNR=1 dB, the constellations of points corresponding to individual signals are well separable, which should result in the effective operation of classification algorithms. In the case of SNR=−1 dB, a significant relocation of several points can be observed, which may cause deterioration of the separation and thus of the quality of the classification. For SNR=−5 dB, the spaces of the individual coefficients overlap, which may result in the incorrect classification.

**Figure 10:**
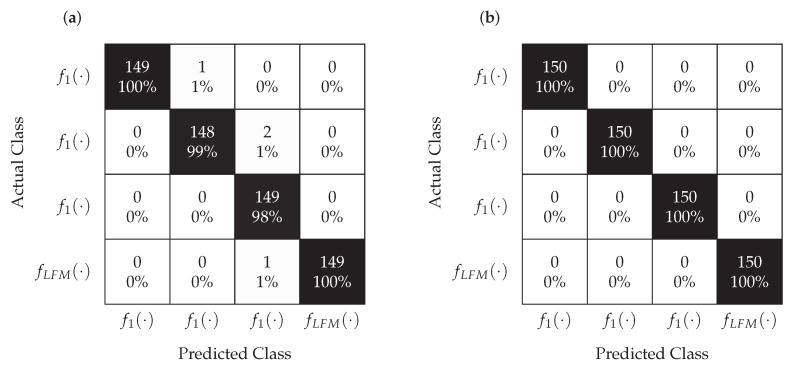
Confusion matrices for the LVQ (**a**) and MLP (**b**) classifiers obtained for SNR=−1 dB.

**Figure 11:**
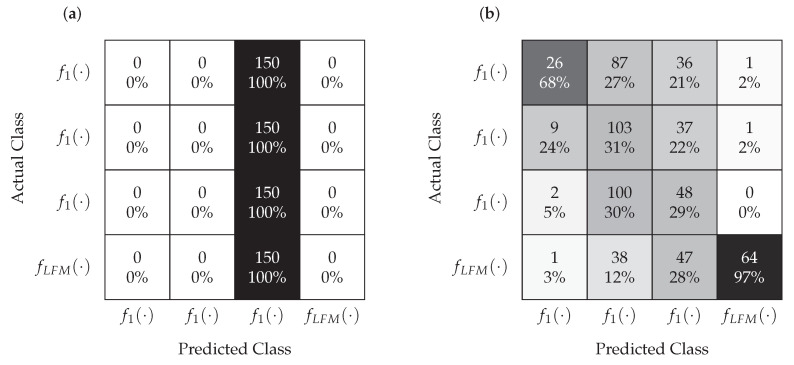
Confusion matrices for the LVQ (**a**) and MLP (**b**) classifiers obtained for SNR=−5 dB.

The classification task was addressed using LVQ and MLP neural networks. The tests were carried out at different SNRs. In each case, $N_{sr}=500$ Monte Carlo simulations were carried out for each signal. The $N_{l}=1400$ realizations constituted a training set and the remaining $N_{t}=600$ realizations were used to test the classifiers. Figure 10 and Figure 11 show the confusion matrices for MLP and LVQ classifiers obtained for SNR=−1 dB and SNR=−5 dB, respectively.

According to the analysis of the confusion matrix presented in Figure 10, for SNR=−1 dB, the classification is correct in the case of the MLP classifier and slightly worse for the LVQ. This means that even the significant relocation of points corresponding to the individual signals, which is visible in Figure 9, does not affect the proper classification performed by classifiers. However, as it results from the analysis of the confusion matrix shown in Figure 11, in the case of SNR=−5 dB, the classification is not correct for both MLP and LVQ. This result corresponds to the results shown in Figure 9, where for this SNR the constellations of points overlap.

**Figure 12:**
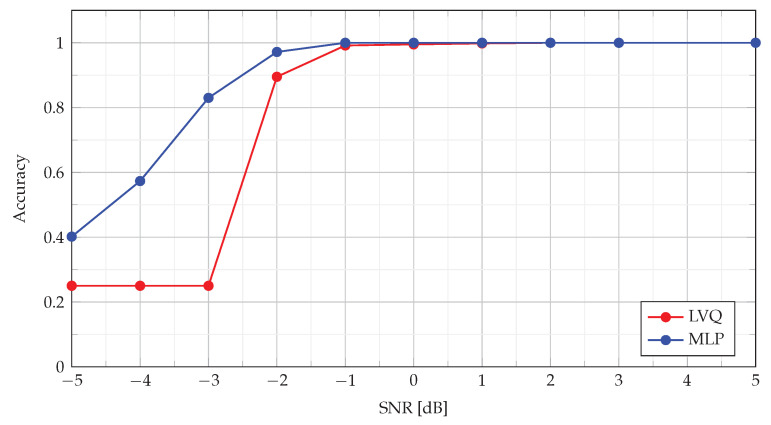
Accuracy (42) metrics of MLP and LVQ classifiers.

The quality of the MLP and LVQ classifiers was also analyzed with the use of ACC metrics. The results for −5 dB ≤SNR≤5 dB are shown in Figure 12.

As can be seen in Figure 12, both classifiers for SNR≥−1 dB provide almost 100% correct identifications of signals. In the case of SNR=−2 dB, the classification accuracy slightly decreases, with MLP being a more effective classifier. For SNR below −2 dB, the classification quality for the MLP algorithm gradually decreases, while in the case of LVQ, the decisions are made completely randomly.

**Figure 13:**
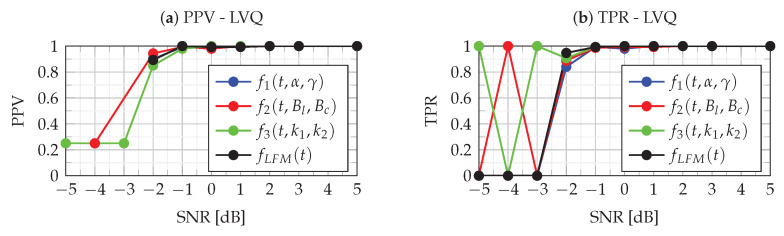
PPV (**a**) and TPR (**b**) metrics of the LVQ classifier.

**Figure 14:**
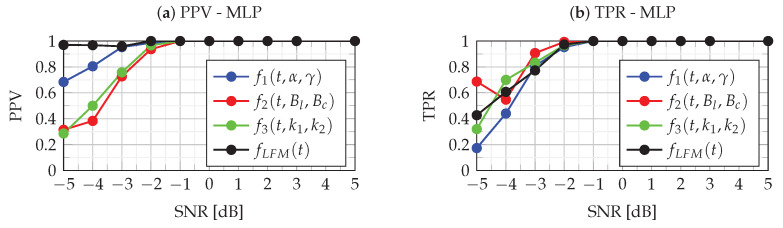
PPV (**a**) and TPR (**b**) metrics of the MLP classifier.

The ACC metrics assess the overall quality of classification without being able to evaluate the identification ability of each class. In this case, PPV and TPR metrics can be used. For multiclass classification, the PPV for each class is the ratio of a correctly predicted class to all predicted classes, while TPR is defined as the ratio of a correctly predicted class to all true class values. Figure 13 and Figure 14 show the PPV and TPR metrics for four classes for MLP and LVQ classifiers. When analyzing the results of the PPV metrics, it should be kept in mind that, in case of the absence of recognition of a given class (both TP and FP), according to definition (43), the PPV value is undefined, and therefore there are missing points in the figure.

The analysis of Figure 13 and Figure 14 allows for assessing the quality of the MLP and LVQ classifiers. The quality of the classifier is indicated by the combined analysis of PPV and TPR values. As can be seen in these figures, in the case of SNR≥−1 dB, both PPV and TPR for all classes take values approximately equal to one, which means that errors of type I and type II are at a minimum level. This means that the identification of each class for SNR≥−1 dB is very good for both MLP and LVQ classifiers. As can be seen in the figures, for the SNR=−2 dB, the classifiers’ performance breaks down. In this case, for the MLP classifier, the PPV and TPR metrics take values in the range of <0.937,1> while for LVQ slightly less, i.e., <0.84,0.947>. This means a slight decrease in the quality of the classification, while the MLP classifier is slightly better. In the case of SNR≤−3 dB, the quality of both classifiers is slightly degraded. In this case, as can be seen in Figure 8 for the LVQ classifier, there is a significant dispersion of type I and type II errors, which means favoring certain classes. However, in the case of MLP (Figure 14), type I and type II errors are at a similar level, and the predictions of classes are more uniform. The incorrect classification results from errors in the estimation of parameters $a_{\phi 2}$, $a_{\phi 4}$ and $a_{\phi 6}$ the determination of which depends on the accuracy of the IFR estimation (23). Due to noise, the location of the maximum CPF obtained for IFR_0_ for noiseless NLFM is shifted to a new random location:

(45) $$\hat{IFR}=IFR_{0}+\delta IFR$$

It should be emphasized that, for SNR≥−1 dB, which is important for practical applications, the classification is error-free for the analyzed classifiers. Classification errors appear for SNR≤−2 dB. Due to the gradual shifting and overlapping of the $a_{\phi 2}$, $a_{\phi 4}$ and $a_{\phi 6}$ parameter space, visible in Figure 9, classification becomes problematic. An additional source of error is the use of the polynomial approximation of the NLFM functions proposed for classification, as discussed in Section 3.

## 6. Conclusions

In this paper, estimation and classification of NLFM signals based on the time–chirp representation have been presented. The majority of useful nonlinearities can be defined or successfully approximated by the polynomial form. However, NLFM radar signals, with the desired PSD and desired AC, require a sufficiently high order of the polynomial for representation of phase and frequency functions. Simulation experiments have shown that CPF is an efficient method of obtaining a polynomial approximation of a phase function.

Having had the coefficients of the phase polynomial from the CPF, the coefficients of the frequency polynomial can be calculated. Based on this relation, the IF estimation of considered NLFM signals is proposed. The simulation results revealed a slightly higher estimation MSE for the proposed CPF-based compared to the QML for NLFM signals. Due to much lower computational load of the proposed approach, the CPF-based method might be preferred in real-time applications.

The promising idea for classification of signals with nonlinear frequency modulations is to use the CPF distribution for the extraction of distinctive features. The modification of the CPF algorithm based on the nonuniform sampling is used for estimation of phase polynomial coefficients for a polynomial of the sixth order. Such calculated coefficients constitute distinctive features for classification.

The classification process has been successfully performed by the LVQ and MLP classifiers, which have the ability to process nonlinear models. Simulations were carried out for different SNRs. The training and classification tasks were performed without the preliminary noise reduction process. This means that classifiers were trained on data with noise that significantly distorted a classified signal, especially for SNR<−1. This results in an unacceptable decrease in the effectiveness of the classification. PPV and TPR measures were successfully used to assess classification results. The results of the research showed that the proposed approach allows for classifying four classes: three considered NLFM signals and an LFM signal. It should be emphasized that, for SNR≥−1 dB, which is important for practical applications, the obtained classification is error-free for the analyzed classifiers. The proposed classification process can be easily extended to more than four classes.

## Data Availability

Not applicable.
